# Acute and Chronic Kidney Dysfunction and Outcome After Stroke Thrombectomy

**DOI:** 10.1007/s12975-020-00881-2

**Published:** 2021-01-04

**Authors:** Simon Fandler-Höfler, Balazs Odler, Markus Kneihsl, Gerit Wünsch, Melanie Haidegger, Birgit Poltrum, Markus Beitzke, Hannes Deutschmann, Christian Enzinger, Alexander R Rosenkranz, Thomas Gattringer

**Affiliations:** 1 Department of Neurology, Medical University of Graz, Auenbruggerplatz 22, 8036 Graz, Austria; 2 Division of Nephrology, Department of Internal Medicine, Medical University of Graz, Graz, Austria; 3 Institute for Medical Informatics, Statistics and Documentation, Medical University of Graz, Graz, Austria; 4 Division of Neuroradiology, Vascular and Interventional Radiology, Department of Radiology, Medical University of Graz, Graz, Austria

**Keywords:** Stroke, Ischemic stroke, Mechanical thrombectomy, Endovascular therapy, Acute kidney injury, Chronic kidney disease

## Abstract

Data on the impact of kidney dysfunction on outcome in patients with stroke due to large vessel occlusion are scarce. The few available studies are limited by only considering single kidney parameters measured at one time point. We thus investigated the influence of both chronic kidney disease (CKD) and acute kidney injury (AKI) on outcome after mechanical thrombectomy. We included consecutive patients with anterior circulation large vessel occlusion stroke receiving mechanical thrombectomy at our center over an 8-year period. We extracted clinical data from a prospective registry and investigated kidney serum parameters at admission, the following day and throughout hospital stay. CKD and AKI were defined according to established nephrological criteria. Unfavorable outcome was defined as scores of 3–6 on the modified Rankin Scale 3 months post-stroke. Among 465 patients, 31.8% had an impaired estimated glomerular filtration rate (eGFR) at admission (< 60 ml/min/1.73 m^2^). Impaired admission eGFR was related to unfavorable outcome in univariable analysis (*p* = 0.003), but not after multivariable adjustment (*p* = 0.96). Patients frequently met AKI criteria at admission (24.5%), which was associated with unfavorable outcome in a multivariable model (OR 3.03, 95% CI 1.73–5.30, *p* < 0.001). Moreover, patients who developed AKI during hospital stay also had a worse outcome (*p* = 0.002 in multivariable analysis). While CKD was not associated with 3-month outcome, we identified AKI either at admission or throughout the hospital stay as an independent predictor of unfavorable prognosis in this study cohort. This finding warrants further investigation of kidney–brain crosstalk in the setting of acute stroke.

## Introduction

Mechanical thrombectomy (MT) has become the primary treatment option for stroke due to large vessel occlusion in the anterior cerebral circulation [1]. However, despite successful recanalization rates of up to 90%, approximately half of the patients treated with MT still have an unfavorable outcome [1, 2]. Determinants of poor prognosis are therefore of great interest. Various pre-treatment factors have been associated with worse outcomes, including higher age, pre-stroke disability, pre-existing vascular brain damage, and comorbidities such as chronic heart disease or diabetes [2, 3].

So far, only few studies have investigated whether impaired kidney function at admission would be associated with worse outcome after MT, yielding rather diverging results. While two of them reported worse functional outcome in patients with a baseline estimated glomerular filtration rate (eGFR) below 60 ml/min/1.73 m^2^, two others found no difference in functional neurological outcome using this eGFR dichotomization [4–7]. Altogether, these studies are limited by the sole investigation of kidney function at hospital admission and therefore were not able to distinguish between patients with chronic kidney disease (CKD) or acute kidney injury (AKI), the latter representing a more dynamic and potentially reversible condition.

As such, longitudinal investigations of eGFR have rarely been performed in stroke populations. Furthermore, the impact of AKI on complications and outcome after acute stroke has not yet been investigated in depth, especially not after MT. Large American retrospective cohort studies found that AKI was associated with increased mortality in stroke patients, but only used ICD-9 coding definitions of AKI, which likely led to an underestimation of the real incidence of AKI [8, 9].

It is a common clinical observation that stroke patients demonstrate a decreased eGFR in the acute phase. This may simply be a consequence of short-term pathophysiological changes such as intermittent hypovolemia, and kidney function would then often revert to normal in the following days. Notably, recent studies found an association even between short periods of kidney dysfunction and adverse effects on remote organs, including pathways such as increased vascular permeability, transcriptomic changes, pro-inflammatory cytokine release, and apoptosis [10, 11]. Despite these findings, studies investigating the relationship between AKI and clinical outcome in stroke patients with MT are limited.

This is why we sought to study the influence of both CKD and AKI on functional outcome in a cohort of consecutive stroke patients treated with MT, by serially exploring serum markers of kidney function throughout the entire hospital stay.

## Methods

### Study Cohort

Using the prospective thrombectomy registry established at our center, we identified all consecutive patients treated with MT for anterior circulation large vessel occlusion stroke (occlusion of the internal carotid artery and/or the proximal middle cerebral artery) between January 2011 and April 2019 [12]. During the entire study period, MT was routinely performed by interventional radiologists using stent retrievers and/or aspiration techniques. A follow-up examination at 3 months was routinely performed during an in-person visit at our stroke outpatient clinic. If this was not possible, a telephone interview was performed.

We excluded patients with missing follow-up information at 3 months (*n* = 3). Clinical and radiological data were prospectively collected; laboratory parameters were additionally exported through the electronic hospital information system, which is used in all hospitals providing acute stroke care in our catchment area.

### Data Assessment

Laboratory parameters were obtained at the initial presentation at the emergency department (either in our center or in external referring hospital), in the morning of the following day after admission (day 1), where a uniform laboratory examination is performed at our center, and throughout the entire hospital stay (up to a maximum of 30 days). Therefore, kidney parameters including serum creatinine and eGFR values were available at initial presentation, day 1, and multiple additional time points throughout the hospital stay whenever indicated in all patients (mean number of creatinine measurements per patient: 5.8 ± 4.3).

Kidney function defined by eGFR was calculated using the well-established CKD-EPI Creatinine Equation [13]. CKD was defined according to the established Kidney Disease Improving Global Outcomes (KDIGO) criteria using the best eGFR measured throughout the hospital stay [14]. As we did not have two separate measurements of albuminuria available and because albuminuria assessment is less reliable in acute medical diseases especially in patients requiring a bladder catheter, we did not categorize CKD regarding that factor. AKI was also defined according to the KDIGO criteria as an increase of serum creatinine of ≥ 1.5 times compared to baseline (or ≥ 0.3 mg/dl within 48 h) [15]. As pre-morbid creatinine values are not available in study cohorts investigating acute medical events such as stroke, we defined the best individual serum creatinine value measured throughout the hospital stay as “baseline” according to the AKI criteria. Therefore, we were able to identify both patients with AKI at admission (initially presenting with creatinine values ≥ 1.5 times compared to improved levels during the hospital stay) or AKI throughout the hospital stay (showing an increase of creatinine values ≥ 1.5 times or ≥ 0.3 mg/dl within 48 h compared to baseline values).

We chose functional neurological outcome defined by the modified Rankin Scale (mRS) at 3 months post-stroke as the primary target variable, dichotomized into favorable outcome (scores of 0–2) and unfavorable outcome (scores of 3–6). We also evaluated mRS values at 3 months on an ordinal scale, the rate of symptomatic intracranial hemorrhage, and mortality 3 months post-stroke. Parenchymal hemorrhage and hemorrhagic infarction were defined according to the established ECASS (European Cooperative Acute Stroke Study) criteria [16].

### Statistical Analysis, Ethical Approval

Statistical analysis was performed using IBM SPSS Statistics for Windows, version 25 (IBM Corp, Armonk, NY, USA). The distribution of continuous variables was evaluated with the Kolmogorov–Smirnov test and histograms. Normally distributed continuous variables were compared by the unpaired Student *t* test; for other distributions, non-parametric tests such as the Mann–Whitney *U* test were used. Categorical variables were investigated using Pearson’s chi-squared test. A binary multivariable regression model for predictors of favorable outcome at 3 months was calculated entering all baseline variables with *p* < 0.10 in the univariable analysis. *p* values of less than 0.05 were considered statistically significant and reported in italics in the tables.

The study was approved by the ethics committee of the Medical University of Graz.

## Results

### Patient Characteristics and Outcome

The study cohort consisted of 465 consecutive patients who had received MT for large vessel occlusion of the anterior cerebral circulation (mean age 68.9 years, 50.5% male). The most prevalent stroke risk factor was arterial hypertension (69.7%), followed by atrial fibrillation (41.7%). Median NIHSS at admission was 15 (interquartile range 11–18); 55.9% of patients had been additionally treated with intravenous thrombolysis. The mean time from stroke symptom onset to groin puncture was 200 min, with successful recanalization in 87.5% of patients (Table 1).

**Table 1:** Clinical characteristics of the study cohort categorized by the 3-month outcome

	Total cohort *n* = 465	mRS 0–2 *n* = 197 (42.4%)	mRS 3–6 *n* = 268 (57.6%)	*p* value Univariable	*p* value Multivariable
Clinical data					
Age (years, mean ± SD)	68.9 ± 13.4	64.3 ± 13.55	72.3 ± 12.3	*< 0.001*	
Male sex	235 (50.5%)	104 (52.8%)	131 (48.9%)	0.41	
Arterial hypertension	324 (69.7%)	112 (56.9%)	212 (79.1%)	*< 0.001*	
Dyslipidemia	104 (22.4%)	41 (20.8%)	63 (23.5%)	0.49	
Chronic heart disease	94 (20.2%)	30 (15.2%)	64 (23.9%)	*0.02*	
Diabetes mellitus	79 (17.0%)	22 (11.2%)	57 (21.3%)	*0.004*	
Atrial fibrillation	194 (41.7%)	64 (32.5%)	130 (48.5%)	*0.001*	
Pre-stroke mRS (median, range)	0 (0–4)	0 (0–2)	0 (0–4)	*< 0.001*	
NIHSS at admission (median, IQR)	15 (11–18)	13 (9–16)	16 (13–19)	*< 0.001*	
MCA/M1 occlusion	300 (64.5%)	129 (65.5%)	171 (63.8%)	0.71	
MCA/M2 occlusion	59 (12.7%)	33 (16.8%)	26 (9.7%)	*0.02*	
Intracranial ICA occlusion	96 (20.6%)	31 (15.7%)	65 (24.3%)	*0.03*	
Acute stroke treatment					
Intravenous thrombolysis	260 (55.9%)	120 (62.8%)	140 (54.9%)	0.09	
Symptom onset to groin puncture (min, median, IQR)	200 (158–247)	200 (158–244)	200 (157–252)	0.84	
Successful recanalization	407 (87.5%)	191 (97.0%)	216 (80.6%)	*< 0.001*	
Kidney parameters					
Creatinine at admission (mg/dl, mean ± SD)	1.06 ± 0.65	0.99 ± 0.46	1.11 ± 0.76	0.20	0.89
Creatinine at day 1 (mg/dl, mean ± SD)	0.96 ± 0.62	0.89 ± 0.50	1.01 ± 0.69	*0.03*	0.95
eGFR at admission (ml/min/1.73 m^2^, mean ± SD)	71.2 ± 24.1	74.8 ± 21.8	68.6 ± 25.4	*0.006*	0.40
eGFR at day 1 (ml/min/1.73 m^2^, mean ± SD)	78.3 ± 24.7	83.7 ± 21.7	74.4 ± 26.1	*< 0.001*	0.74
eGFR < 60 ml/min/1.73 m^2^ at admission	148 (31.8%)	48 (24.4%)	100 (37.3%)	*0.003*	0.96
CKD G3 or worse	55 (11.8%)	19 (9.6%)	36 (13.4%)	0.21	0.33
AKI at admission	114 (24.5%)	27 (13.7%)	87 (32.5%)	*< 0.001*	*< 0.001*
AKI during the hospital stay	85 (18.3%)	21 (10.7%)	64 (23.9%)	*< 0.001*	*0.002*

*AKI* acute kidney injury, *CKD* chronic kidney disease, *eGFR* estimated glomerular filtration rate,  *ICA *internal carotid artery*, MCA *middle cerebral artery*, mRS* modified Rankin Scale, *NIHSS* National Insitutes of Health Stroke Scale

A total of 197 patients (42.4%) had favorable outcome (mRS 0–2) at 3 months post-stroke. The baseline variables age, arterial hypertension, chronic heart disease, diabetes mellitus, atrial fibrillation, pre-stroke mRS, NIHSS at admission, occlusion location, and unsuccessful recanalization were associated with unfavorable outcome in univariable analyses (Table 1).

### Kidney Function Parameters, Chronic Kidney Disease

At hospital admission, mean serum creatinine was 1.06 ± 0.65 mg/dl and mean eGFR was 71.2 ± 24.1 ml/min/1.73 m^2^. On average, kidney parameters slightly improved at day 1 (mean serum creatinine 0.96 ± 0.62 mg/dl, mean eGFR 78.3 ± 24.7 ml/min/1.73 m^2^). While 148 (31.8%) patients had an eGFR < 60 ml/min/1.73 m^2^ at admission, over time kidney parameters improved in most patients—only 55 (11.8%) had CKD G3a or worse (indicating eGFR values consistently < 60 ml/min/1.73 m^2^) during the entire hospital stay (Table 1).

While absolute serum creatinine values at day 1 and eGFR at admission and day 1 were associated with unfavorable outcome at 3 months in univariable analyses, all these variables lost significance in multivariable analysis adjusting for baseline factors related to outcome in univariable analysis (age, hypertension, chronic heart disease, diabetes, atrial fibrillation, NIHSS at admission, pre-stroke mRS, occlusion location, intravenous thrombolysis, and successful recanalization; Table 1). All of the mentioned kidney parameters were not associated with symptomatic intracranial hemorrhage (*p* > 0.1, data not shown).

### Acute Kidney Injury at Hospital Admission

**Fig. 1:**
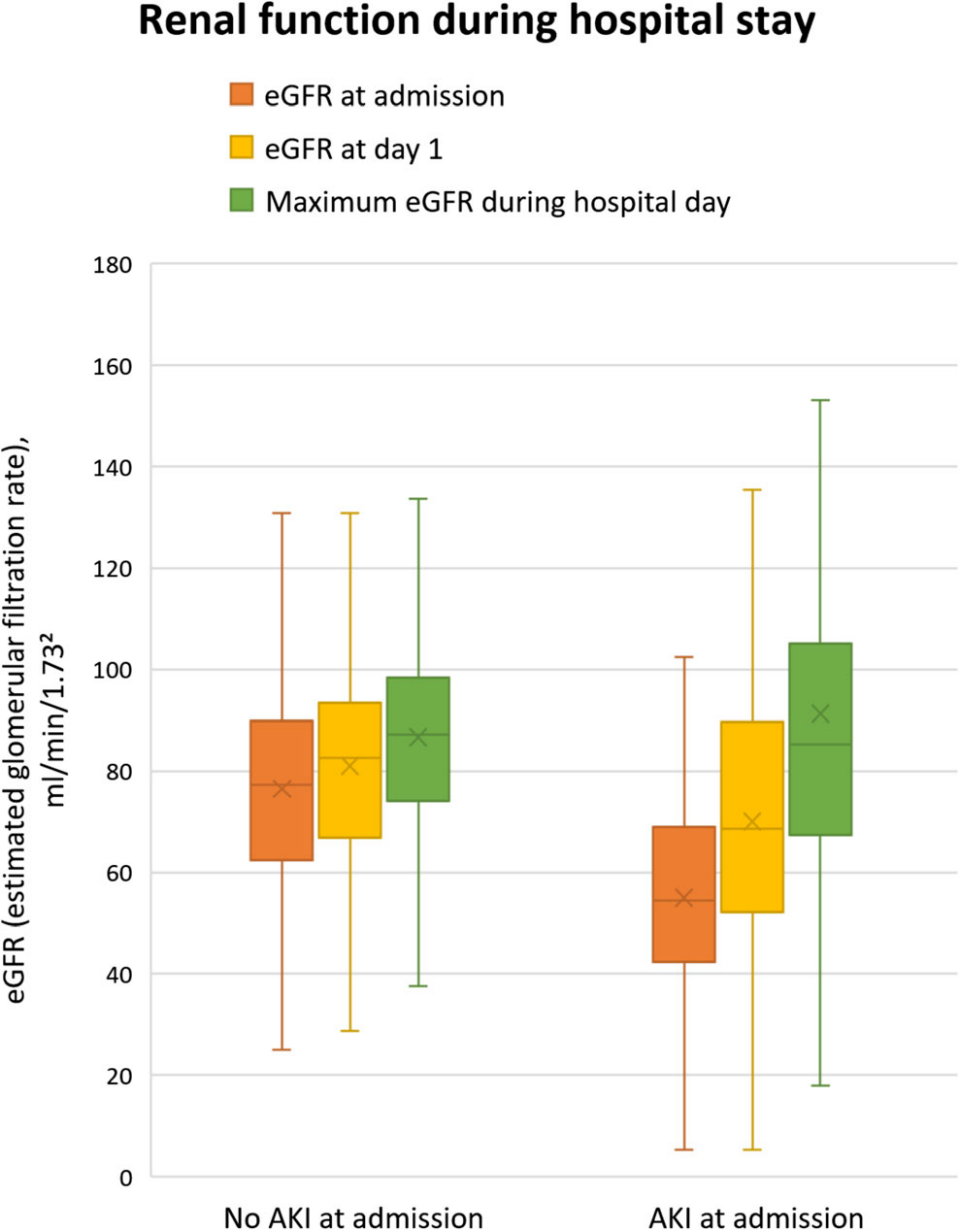
Box-and-whisker plot comparing patients with and without acute kidney injury (AKI) at admission regarding kidney function during the hospital stay (horizontal lines representing median values, boxes spanning the interquartile range, vertical lines showing maximum and minimum values)

AKI at admission was present in 114 patients (24.5%) and predominantly mild; the majority of affected patients (102 of 114) met criteria for stage 1 AKI (defined as a rise of 1.5 to 2.0 times of baseline serum creatinine). Figure 1 shows a comparison of the course of kidney function in patients with AKI versus those without.

AKI at hospital admission was strongly associated with unfavorable outcome in univariable analysis, and this association remained significant in multivariable analysis (odds ratio 3.03, 95% CI 1.73–5.30, *p* < 0.001; Table 2). The other baseline variables predicting unfavorable outcome in this multivariable model were higher age, higher pre-stroke mRS, higher NIHSS at admission, and unsuccessful recanalization.

**Table 2:** Multivariable regression model regarding unfavorable neurological outcome (mRS 3–6) at 3 months post-stroke

Variable	Odds ratio	95% confidence interval	*p* value
Age (per year)	1.04	1.02–1.06	*< 0.001*
Pre-stroke mRS (per point)	1.47	1.04–2.06	*0.03*
Arterial hypertension	1.45	0.87–2.56	0.15
Chronic heart disease	1.24	0.69–2.22	0.48
Diabetes mellitus	1.25	0.66–2.38	0.50
Atrial fibrillation	1.23	0.76–1.99	0.39
NIHSS at admission (per point)	1.15	1.09–1.22	*< 0.001*
MCA/M2-occlusion	0.97	0.49–1.93	0.94
Intracranial ICA occlusion	1.66	0.93–2.99	0.09
Intravenous thrombolysis	0.75	0.47–1.21	0.24
Successful recanalization	0.10	0.04–0.27	*< 0.001*
Acute kidney injury at presentation	3.03	1.73–5.30	*< 0.001*

When comparing patients with AKI at hospital admission to those without, AKI patients had more comorbidities and significantly worse kidney function parameters. There were no differences in age, sex, or stroke treatment (Table 3). Symptomatic intracranial hemorrhage was not associated with AKI at admission, but patients with AKI at admission tended to show higher rates of hemorrhagic infarction or parenchymal hemorrhage (27.2% vs. 18.8%, *p* = 0.05; Table 3).

**Table 3:** Comparison of patients with and without acute kidney injury at hospital admission

	AKI at admission	No AKI at admission	p value
	*n* = 114	*n* = 351	
Clinical data			
Age (years, mean ± SD)	69.5 ± 14.7	68.7 ± 13.0	0.57
Male sex	46 (43.0%)	178 (52.4%)	0.09
Arterial hypertension	88 (77.2%)	236 (67.2%)	*0.04*
Dyslipidemia	28 (24.6%)	76 (21.7%)	0.52
Chronic heart disease	31 (27.2%)	63 (17.9%)	*0.03*
Diabetes mellitus	26 (22.8%)	53 (15.1%)	0.06
Atrial fibrillation	52 (45.6%)	143 (40.5%)	0.33
Pre-stroke mRS (median, range)	0 (0–4)	0 (0–4)	0.63
NIHSS at admission (median, IQR)	15 (12–19)	14 (11–18)	0.09
MCA/M1 occlusion	82 (71.9%)	218 (62.1%)	0.06
MCA/M2 occlusion	9 (7.9%)	50 (14.2%)	0.08
Intracranial ICA occlusion	21 (18.4%)	75 (21.4%)	0.50
Acute stroke treatment			
Intravenous thrombolysis	56 (52.8%)	204 (60.0%)	0.19
Time to groin puncture (minutes, median, IQR)	195 (150–250)	200 (156–247)	0.72
Successful recanalization	95 (84.8%)	308 (88.8%)	0.27
Kidney parameters			
Creatinine at admission (mg/dl, mean ± SD)	1.45 ± 1.14	0.93 ± 0.27	*< 0.001*
Creatinine at day 1 (mg/dl, mean ± SD)	1.20 ± 1.13	0.88 ± 0.26	*0.003*
Highest creatinine within 30 days post-stroke (mg/dl, mean ± SD)	1.51 ± 1.25	1.03 ± 0.37	*< 0.001*
Lowest creatinine within 30 days post-stroke (mg/dl, mean ± SD)	0.95 ± 0.93	0.82 ± 0.26	*0.05*
eGFR at admission (ml/min/1.73 m^2^, mean ± SD)	55.0 ± 22.9	76.5 ± 22.1	*< 0.001*
eGFR at day 1 (ml/min/1.73 m^2^, mean ± SD)	70.0 ± 28.6	81.0 ± 22.7	*< 0.001*
CKD G3 or worse	21 (18.4%)	34 (9.7%)	*0.01*
Outcome parameters			
Symptomatic intracranial hemorrhage	6 (5.6%)	9 (2.6%)	0.14
Hemorrhagic infarction or parenchymal hemorrhage	31 (27.2%)	66 (18.8%)	0.05
mRS 3 months post-stroke (median, IQR)	4 (3–5)	3 (1–5)	*< 0.001*
Favorable outcome at 3 months (mRS 0–2)	27 (23.7%)	170 (48.4%)	*< 0.001*

*AKI* acute kidney injury, *CKD* chronic kidney disease, *eGFR* estimated glomerular filtration rate, *ICA* internal carotid artery, *MCA* middle cerebral artery, *mRS* modified Rankin Scale, *NIHSS* National Insitutes of Health Stroke Scale

### Acute Kidney Injury During the Hospital Stay

When investigating the occurrence of AKI during the hospital stay (as opposed to AKI at hospital admission), we found that an additional 85 patients (18.3%) developed AKI meeting the applied criteria (Table 1). AKI occurrence over time in the hospital was also related to the unfavorable 3-month outcome (*p* < 0.001 in univariable, *p* = 0.002 in multivariable analysis; odds ratio 2.64, 95% CI 1.41–4.94). Interestingly, AKI during hospital stay and AKI at admission were not associated with each other (*p* = 0.38), implying that these subgroups represent different entities.

Only five patients had an early increase of serum creatinine ≥ 0.3 mg/dl between hospital admission and day 1, potentially indicating contrast-associated nephropathy.

## Discussion

We here performed a comprehensive analysis of routine serum biomarkers of kidney function in a comparatively large cohort of consecutive patients treated with MT for anterior circulation large vessel occlusion stroke at a single center. While eGFR and serum creatinine at hospital admission and day 1 correlated with functional outcome in univariable analysis, no significant effect remained in a multivariable model adjusted for other well-established prognostic factors. However, and most importantly, patients with AKI either at initial presentation or developing it throughout the hospital stay had worse outcomes 3 months post-stroke in multivariable analysis.

Previous studies investigating kidney function in stroke patients mostly focused on eGFR values at admission alone and limited their analysis to a dichotomization of eGFR into values below or above 60 ml/min/1.73 m^2^ [4–7], obviating a differentiation between pre-existing CKD or AKI. However, these represent two entities, which constitute rather different clinical situations and consequences.

Contrary to our results, a recent Chinese study found (slightly) worse outcomes in stroke patients treated with MT who had an eGFR < 60 ml/min/m^2^ at admission using multivariable models [4]. While the rate of such a reduced eGFR at admission was significantly higher in our patients (31.8% compared to 15.8%), numerous other significant heterogeneities exist between these studies, including differences in demographics, underlying vascular risk factors and stroke etiologies, much faster recanalization times in our study, and ethnic differences, rendering a direct comparison difficult. Two other cohort studies also did not find an effect of baseline kidney function on the 3-month functional outcome in stroke patients treated with MT, while a fourth one did [5–7].

Aside from investigating kidney parameters at baseline, our study expands on multiple measurements of kidney function, thus allowing the important differentiation between CKD and AKI as mentioned before. We found that our MT patients frequently met criteria for AKI at admission (approximately one quarter of our study cohort was affected), and even though it was mostly mild (AKI stage 1), it was independently associated with a worse 3-month outcome. Predictors for AKI at admission included several comorbidities such as hypertension and chronic heart disease, as well as generally worse kidney function. Importantly, there were no differences in age or stroke treatment times. Dehydration is likely an important factor for the development of AKI pre-stroke, but infections or acute-on-chronic conditions such as decompensated heart failure in the setting of acute stroke leading to cardio-renal syndrome may also be potential trigger factors for AKI in the acute phase of a cerebrovascular event [17].

AKI is known to cause increased plasma levels of inflammatory mediators including tumor necrosis factor alpha, interleukins 1 and 6, and interferon-γ, causing systemic inflammation, oxidative stress, and endothelial dysfunction [11, 18, 19]. These factors lead to activation of microglia and impairment of the blood–brain barrier, which are seen as the main cornerstones of neuroinflammation in patients with AKI [20]. Additional potentially deleterious consequences of AKI include derangement of neurotransmitters and infiltration of neurotoxic metabolites in the brain due to disruption of the blood–brain barrier integrity [21, 22].

Furthermore, activation of the innate immune system during AKI through toll-like receptors may contribute to neuronal injury during ischemia-reperfusion injury [20, 23]. Aquaporins are also believed to be involved in AKI-associated brain injury, as inflammatory cytokines mediate Aquaporin-4 expression on astrocytes, potentially contributing to cerebral edema [20]. Animal models of AKI have shown cerebral inflammation and blood–brain barrier disruption causing functional brain changes, such as neuronal microgliosis in the cortex and especially in the hippocampus [24]. All these factors could be especially critical in the setting of a cerebral large vessel occlusion and subsequent reperfusion, likely leading to increased transient and permanent loss of brain tissue and function.

AKI can also cause cardiac injury and arrhythmia including atrial fibrillation [11, 25] and may be therefore not only a factor associated with worse outcome in stroke patients, but also a trigger for stroke itself. Our results correspond to a recent study of ischemic stroke patients treated with intravenous thrombolysis, which reported that dynamic changes in eGFR were associated with higher 3-month mortality [26].

Additionally, we were also able to show that AKI developing during the hospital stay was independently associated with worse outcome. This is not surprising, as AKI is a known important complication of vascular events such as myocardial infarction and stroke and is also associated with increased morbidity and mortality [27]. Similar findings as ours were reported in previous studies. In this context, a review reported a pooled incidence rate of AKI after admission for stroke of 9.6%, but this figure is likely underestimated, as most included studies only used retrospective ICD-9/10 coding definitions. The rate of 18.3% that we found in our study was very similar to those in studies included in the review that defined AKI using serum creatinine values (reporting AKI rates of 14.5, 17.9, and 26.7%) [28].

While there was no association between kidney parameters and the rate of symptomatic intracranial hemorrhage, patients with AKI at admission tended to have higher rates of hemorrhagic infarction or parenchymal hemorrhage. We did not see a significant role of contrast-associated nephropathy, similar to a recent study in stroke patients treated with MT and a review of studies investigating ischemic stroke patient who underwent CT angiography and/or CT perfusion [29].

Limitations of our work include the single-center design, making generalization of our findings to other cohorts less reliable. Clinical data including outcome at 3 months was assessed prospectively (blinded to this specific study purpose); only laboratory data were collected retrospectively.

As this was a clinical cohort study, we did not have pre-stroke kidney parameters available for comparison. Instead, we used the best values obtained during the hospital day for definition of both acute and chronic changes in kidney function. This might have led to an underestimation of the rates of AKI and CKD (as patients may sometimes not have reached their “baseline” kidney parameter before discharge). We did not investigate albuminuria, an important marker of kidney dysfunction, which would have required at least two urine samples for adequate validity. Furthermore, we did not include findings from neuroimaging, as available modalities varied within the study cohort. Nevertheless, this is yet the most comprehensive analysis of kidney function in stroke patients with acute intracranial large vessel occlusion.

## Conclusion

In this cohort study of consecutive patients treated with mechanical thrombectomy, we did not find an association between chronic kidney disease and patient outcome in multivariable analysis. However, acute kidney injury either at admission or throughout the hospital stay represented independent predictors of unfavorable outcome 3 months post-stroke. A stronger clinical focus even on mild episodes of acute kidney injury may thus be helpful to potentially mitigate additional harm in affected patients. However, for firmer conclusions, both translational and prospective clinical studies are required to more precisely investigate the kidney–brain crosstalk in stroke patients, especially in the context of acute stroke and large vessel occlusion.

## Data Availability

The datasets generated during this study are available from the corresponding author upon reasonable request.
